# A New Antagonist of *Caenorhabditis elegans* Glutamate-Activated Chloride Channels With Anthelmintic Activity

**DOI:** 10.3389/fnins.2020.00879

**Published:** 2020-08-19

**Authors:** María Julia Castro, Ornella Turani, María Belén Faraoni, Darío Gerbino, Cecilia Bouzat

**Affiliations:** ^1^Departamento de Biología, Bioquímica y Farmacia, Instituto de Investigaciones Bioquímicas de Bahía Blanca (INIBIBB), Universidad Nacional del Sur (UNS)-Consejo Nacional de Investigaciones Científicas y Técnicas (CONICET), Bahía Blanca, Argentina; ^2^Instituto de Química del Sur (INQUISUR), Universidad Nacional del Sur (UNS)-Consejo Nacional de Investigaciones Científicas y Técnicas (CONICET), Bahía Blanca, Argentina

**Keywords:** *C. elegans*, Cys-loop receptors, glutamate-activated chloride channels, patch-clamp, anthelmintic

## Abstract

Nematode parasitosis causes significant mortality and morbidity in humans and considerable losses in livestock and domestic animals. The acquisition of resistance to current anthelmintic drugs has prompted the search for new compounds for which the free-living nematode *Caenorhabditis elegans* has emerged as a valuable platform. We have previously synthetized a small library of oxygenated tricyclic compounds and determined that dibenzo[*b,e*]oxepin-11(6H)-one (doxepinone) inhibits *C. elegans* motility. Because doxepinone shows potential anthelmintic activity, we explored its behavioral effects and deciphered its target site and mechanism of action on *C. elegans.* Doxepinone reduces swimming rate, induces paralysis, and decreases the rate of pharyngeal pumping required for feeding, indicating a marked anthelmintic activity. To identify the main drug targets, we performed an *in vivo* screening of selected strains carrying mutations in Cys-loop receptors involved in worm locomotion for determining resistance to doxepinone effects. A mutant strain that lacks subunit genes of the invertebrate glutamate-gated chloride channels (GluCl), which are targets of the widely used antiparasitic ivermectin (IVM), is resistant to doxepinone effects. To unravel the molecular mechanism, we measured whole-cell currents from GluClα1/β receptors expressed in mammalian cells. Glutamate elicits macroscopic currents whereas no responses are elicited by doxepinone, indicating that it is not an agonist of GluCls. Preincubation of the cell with doxepinone produces a statistically significant decrease of the decay time constant and net charge of glutamate-elicited currents, indicating that it inhibits GluCls, which contrasts to IVM molecular actions. Thus, we identify doxepinone as an attractive scaffold with promising anthelmintic activity and propose the inhibition of GluCls as a potential anthelmintic mechanism of action.

## Introduction

Nematode parasitosis is an important cause of mortality and morbidity in humans and affects livestock and domestic animals. As many as one-third of the world’s population harbors infections with helminths. Also, nematodes have an important negative impact on animal productivity worldwide. The reduced anthelmintic drug development and the ever-increasing resistance of nematodes to the limited number of drugs have become a global concern for veterinary and human health.

Parasitic nematodes are not ideal laboratory animals for drug screening due to the difficulty for genetic manipulation and the need for infected host animals. *C. elegans* shares physiological and pharmacological features with parasitic nematodes and it is sensitive to most anthelmintic drugs. The major neurotransmitter receptors are similar between *C. elegans* and parasitic species (Angstadt et al., 1989; Holden-Dye and Walker, 2007). Thus, the free-living nematode *C. elegans* has contributed as a parasitic model to defining mechanisms of antiparasitic drug action.

Cys-loop receptors are major targets of anthelmintic drugs in parasites and *C. elegans* (Holden-Dye and Walker, 2006; Beech and Neveu, 2015). They belong to the family of pentameric ligand-gated ion channels and play key roles throughout the nervous system in vertebrates and invertebrates. They are involved in physiological processes, including muscle contraction, and are targets for clinically relevant drugs (Wolstenholme, 2011). In vertebrates, Cys-loop receptors include acetylcholine nicotinic (nAChRs) and 5-hydroxytryptamine type 3 (5-HT_3_) receptors, which are cationic channels, and GABA_*A*_ and glycine receptors, which are anionic channels. *C. elegans* and parasitic nematode muscle contains two different types of nAChRs, a levamisole-sensitive (L-AChR) and a nicotine-sensitive (N-AChR), and a GABA receptor. L-AChRs mediate muscle contraction whereas GABA receptors mediate muscle relaxation. These two receptors are essential for the typical sinusoidal movement and are targets of anthelmintic compounds, like levamisole and pyrantel (L-AChR agonists) and piperazine (GABA receptor agonist) (Martin, 1997; Fleming et al., 1997; Culetto et al., 2004; Towers et al., 2005; Bamber et al., 2005; Rayes et al., 2007). N-AChR is a homopentameric receptor that responds to acetylcholine and nicotine and its role in locomotion is not fully understood (Touroutine et al., 2005). Compared to vertebrates, invertebrates contain a larger variety of Cys-loop receptors, including a unique type of glutamate-gated chloride channels (GluCl) (Jones and Sattelle, 2008).

GluCls are of considerable medical and economical importance because they are targets of macrocyclic lactones, such as ivermectin (IVM), which are the most widely used antiparasitic drugs (Chen and Kubo, 2018). IVM is used in veterinary for gastrointestinal roundworms, lungworms, grubs, sucking lice, and mange mites and in humans for treating filarial diseases (Campbell, 2012).

There are six *C. elegans* genes encoding GluCl subunits: *avr-14* (GluClα3 subunit), *avr*-*15* (GluClα2), *glc-1* (GluClα1), *glc-2* (GluClβ), *glc-3* (GluClα4), and *glc-4* (Cully et al., 1994, 1996; Vassilatis et al., 1997; Dent et al., 2000; Horoszok et al., 2001). Functions associated with GluCls include pharyngeal pumping, which is required for feeding and maintaining hydrostatic pressure, and for the regulation of locomotion, and olfactory and temperature responses (Jones and Sattelle, 2008). Heterologous expression studies have shown that both GluClα1 and GluClβ subunits form functional homomeric receptors, the first responding to IVM and the latter to glutamate (Vassilatis et al., 1997; Li et al., 2002), and that GluClα1/β heteropentamers respond to both IVM and glutamate (Dent et al., 1997; Degani-Katzav et al., 2016). The X-ray structure of the homomeric GluClα has revealed information about the binding sites of the allosteric agonist IVM, the orthosteric agonist L-glutamate and the open-channel blocker picrotoxin (Hibbs and Gouaux, 2011).

We have recently synthetized a series of oxygenated tricyclic compounds and determined their anthelmintic activity by measuring rapid effects on *C. elegans*. The exposure to dibenzo[*b,e*]oxepin-11(6H)-one (doxepinone) produced a rapid concentration-dependent decrease of the thrashing rate (IC_50_ ∼300 μM), which is a measure of *C. elegans* motility and swimming rate (Buckingham and Sattelle, 2009; Scoccia et al., 2017). Doxepinone is considered a privileged structure, which refers to compounds whose scaffolds commonly consist of a rigid ring, including heteroring systems that present appended residues in well-defined orientations required for target recognition (Evans et al., 1988). Through appropriate functional group modifications, these scaffolds can provide ligands for a number of functionally and structurally discrete biological receptors, and have, therefore, attracted interest across a broad spectrum of sciences from chemistry and biology to medicine. Because doxepinone is a privileged structure with potential anthelmintic activity, we here explored in detail its behavioral effects and deciphered its target site and mechanism of action on *C. elegans* as a parasite model. We propose doxepinone as an attractive scaffold with potential antiparasitic activity mediated, at least in part, through GluCls.

## Materials and Methods

### Synthesis of Doxepinone

Dibenzo[*b,e*]oxepin-11(6H)-one (named as doxepinone) was synthetized following the protocol developed by our group (Scoccia et al., 2017 and Supplementary Figure S1). Briefly, 2-(phenoxymethyl) benzoic acid was prepared by treating the commercially available isobenzofuran-1(3H)-one with sodium phenoxide, which was obtained by reacting phenol with NaH in the presence of DMF at reflux. 2-(phenoxymethyl) benzoic acid was then cyclized by intramolecular acylation from the carboxylic acid compound by using FeCl_2_ and dichloromethyl methyl ether as cooperative system in the presence of dichloromethane at room temperature. Purity was determined by elemental analysis and melting point.

L-Glutamic acid monosodium salt hydrate, ivermectin and dimethyl sulfoxide were from Sigma-Aldrich Chem Co.

### *Caenorhabditis elegans* Strains and Culture

Nematode strains used were: N2: Bristol wild type; and the null mutants of Cys-loop receptor subunits: RB918: *acr-16(ok789)*; DA1316: *avr-14(ad1302);avr-15(ad1051);glc-1(pk54)*; CB382: *unc-49(e382)*; CB904: *unc-38(e264)*; MT9668: *mod-1(ok103).* All strains were obtained from the *Caenorhabditis* Genetic Center, supported by the National Institutes of Health - Office of Research Infrastructure Programs (P40 OD010440). Nematodes were maintained at 21°C using standard culture methods (Brenner, 1974; Stiernagle, 2006; Hernando et al., 2012, 2019). Assays were carried out following standard protocols described in WormBook^1^.

### Locomotion and Paralysis Assays

All behavioral assays were done at room temperature (21–23°C) and all comparisons were done in parallel. Assays were performed with young adult hermaphrodite worms from synchronized plates. For comparison among different drug concentrations or strains, the assays of the control and different conditions were performed simultaneously. For each condition, 30 worms (*n* = 30) were used in paralysis assays and 20 worms (*n* = 20) in thrashes assays. Each condition was evaluated 4 times in different days with different worm batches and always in parallel with the control, as described before (Hernando et al., 2012, 2019).

Thrashing assays were performed in 100 μl M9 buffer in the absence or presence of the drug in a 96-well microliter plate as described before (Jones et al., 2011). A single thrash was defined as a change in the direction of bending at the mid body. All assays were carried out by two independent operators and were blinded to the sample identities.

Paralysis was determined on agar plates containing the tested drug at room temperature as described before (Hernando et al., 2012). Body paralysis was followed by visual inspection at the indicated time (up to 120 min) and was defined as the lack of body movement in response to prodding.

For prodding, we used the gentle touch stimulus delivered to the body with an eyebrow hair, avoiding touching the animals too near the tip of the head or tail (Hernando et al., 2019). We evaluated different types of nematode paralysis: flaccid paralysis in which worms appear lengthened; spastic paralysis in which worms appear shorter, and stationary paralysis in which worms are immobile but respond to prodding by contracting body wall muscle (Kass et al., 1980; Hernando et al., 2019). Videos were acquired with a digital camera ToupCam UCMOS 05100KPA (Toup Tek Photonics).

Stock solutions of ivermectin (IVM) and doxepinone were in dimethyl sulfoxide (DMSO). For all assays, the final concentration of DMSO was lower than 1%.

### Pharyngeal Pumping Measurements

Measurements were performed with young adult worms on agar plates. Plates contained 1 μM IVM, 50 or 100 μM doxepinone. DMSO was used as the vehicle and was present in all control plates at concentrations lower than 1% v/v. Wild-type and DA1316 young adult worms were transferred to drug plates and allowed to remain at 20°C for a 30 min period. We performed 3 independent whole experiments with different worm batches. Each experiment included comparison of the effects of drugs on wild-type and mutant strains in parallel, with *n* = 14 animals for each condition in each experiment. The number of contractions in the terminal bulb of the pharynx (pumps per minute) was counted using a stereomicroscope at 50× magnification.

### Body Length Measurements

Young adult hermaphrodite worms (*n* = 10 worms for each condition) were transferred to NGM plates containing 2.5 mM doxepinone. Vehicle (1% DMSO) was used as a control. After 2 h, images were acquired with a digital camera ToupCam UCMOS 05100KPA (Toup Tek Photonics) and body length was measured using FIJI-ImageJ software. Four independent whole experiments were analyzed in parallel with the controls.

### Heterologous Cell Expression of GluCls

GluCls were transiently expressed in BOSC 23 cells, which are modified HEK 293T cells (Pear et al., 1993). The cDNAs encoding the *C. elegans* GluClα1 (containing *gfp* between transmembrane domains M3 and M4) and GluClβ subunits, both subcloned into the pcDNA3.1 vector, were kindly provided by Dr. Paas (Degani-Katzav et al., 2016). Cells were transfected by calcium phosphate precipitation with the subunit cDNAs (total 4 μg/35 mm dish) at a ratio GluClα1:GluClβ 1:1 essentially as described before (Bouzat et al., 2008; Nielsen et al., 2018). All transfections were carried out for about 8–12 h in DMEM with 10% fetal bovine serum and were terminated by exchanging the medium. Cells were used for whole-cell recordings 2 or 3 days after transfection, time at which maximal functional expression levels are typically achieved (Bouzat et al., 2008).

### Whole-Cell Recordings From BOSC23 Cells

Macroscopic currents were recorded in the whole-cell configuration as described previously (Bouzat et al., 2008; Corradi et al., 2009).

The pipette was filled with intracellular solution (ICS) containing 134 mM KCl, 5 mM EGTA,1 mM MgCl_2_, and 10 mM HEPES (pH 7.3). The extracellular solution (ECS) contained 150 mM NaCl, 0.5 mM CaCl_2_, and 10 mM HEPES (pH 7.4). After the whole cell formation, ECS containing the agonist or drug was rapidly applied using a three-tube perfusion system with elevated solution reservoirs for gravity-driven flow and switching valves controlled by a VC3 controller (ALA Scientific). The solution exchange time was estimated by the open pipette method as described by Liu and Dilger (1991). This method consists in applying a pulse of 50% diluted ECS to an open patch pipette, which produces a sudden change in the current measured by patch-clamp amplifier. After proper adjustment of the electrode position, the current jump in our system varied between 0.1 and 1 ms (Corradi et al., 2009; Andersen et al., 2016). The compound doxepinone was dissolved in ECS from DMSO stock solutions. The final concentration of DMSO used to solubilize doxepinone was lower than 0.2%. To study the modulatory action of doxepinone, responses were evaluated following co-application or preincubation protocols. After whole cell formation, 3 mM glutamate-elicited currents (control currents) were first recorded by a pulse (6 s) of ECS containing glutamate. The compound was then co-applied with glutamate (Co-application protocol) or applied during 1 min in the absence of glutamate before the second glutamate pulse (Preincubation protocol). For all experiments, the duration of the glutamate pulse was 6 s and the time of recording was 8 s. A 60-s wash with ECS alone allowed total recovery of control currents. The treated currents were normalized to currents elicited by glutamate alone in the same cell (control current). At the end of the protocol, the control current was again tested and the experiments in which the currents were reduced to more than 80% of the original control current were discarded.

Currents were filtered at 5 kHz and digitized at 20 kHz using an Axopatch 200B patch-clamp amplifier (Molecular Devices, CA, United States) and acquired using WinWCP software (Strathclyde Electrophysiology Software, University of Strathclyde, Glasgow, United Kingdom). The recordings were analyzed using the ClampFit software (Molecular Devices, CA, United States). Currents were fitted by a single exponential function according to the equation:

I(t) = I[exp(-t/τ_*d*_)] + I_∞_

in which t is time, I is the peak current, I_∞_ is the steady state current value, and τ_*d*_ is the decay time constant. Net charge was calculated by current integration (Andersen et al., 2016). The rise time (tr_10__–__90__%)_ corresponds to the time taken by the current to increase from 10 to 90% of its maximal value.

### Data and Statistical Analysis

Experimental data are shown as mean ± SD. Statistical comparisons were done using two-tailed Student’s *t*-test for pairwise comparisons or oneway ANOVA followed by Bonferroni’s *post hoc* tests for multiple comparisons. All the tests were performed with SigmaPlot 12.0 (Systat Software, Inc.). Statistically significance was established at *p*-values < 0.05 (^∗^*p* < 0.05, ^∗∗^*p* < 0.01, ^∗∗∗^*p* < 0.001). Concentration–response curves were determined by non-linear regression fits to the Hill equation using Prism 5.0 (GraphPad, San Diego, CA, United States).

## Results

### 1-Screening of *C. elegans* Mutant Strains for Resistance to Dibenzo[*b,e*]oxepin-11(6H)-One (Doxepinone): Measurements of Swimming Rates

We have previously found that dibenzo[*b,e*]oxepin-11(6H)-one, referred to as doxepinone, produced rapid paralysis of *C. elegans* in liquid medium (Scoccia et al., 2017). After 10 min exposure, the compound produced a concentration-dependent decrease of the thrashing rate (IC_50_ ∼300 μM) (Scoccia et al., 2017). Because doxepinone is an interesting synthetic molecule with potential anthelmintic activity we sought to explore its anthelmintic effects and identify target sites.

**FIGURE 1:**
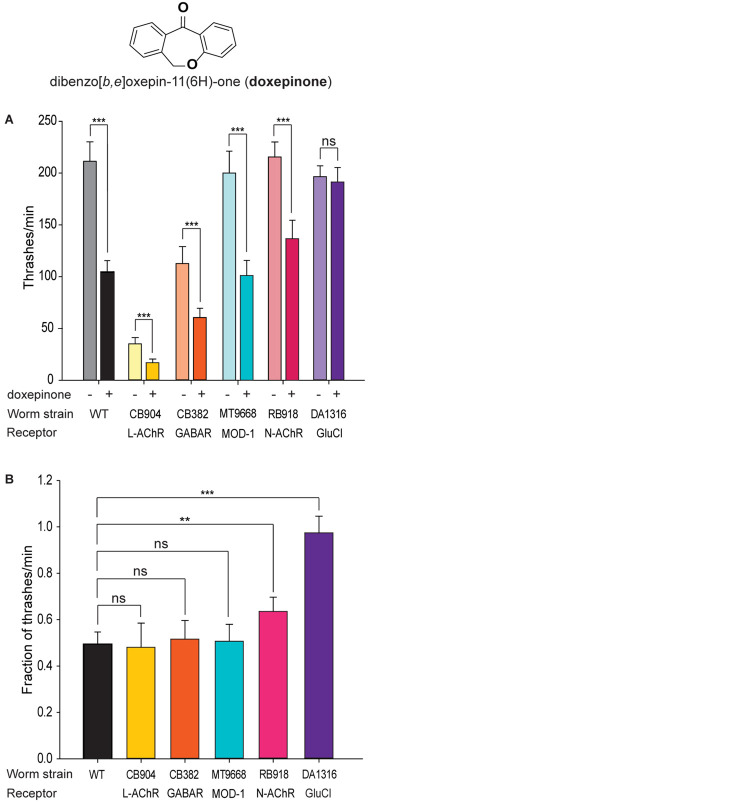
Screening of mutants for resistance to doxepinone. Synchronized young adult wild-type worms were used. Measurements were performed in liquid medium after 30 min incubation in M9 buffer containing 1% DMSO in the absence or presence of 0.1 mM doxepinone. A single thrash was defined as a change in the direction of bending at the mid body. The non-functional receptor in each mutant strain is indicated. *n* = 20 worms per condition, repeated in 4 different days and worm batches, in parallel with the control. **(A)** Bar chart showing the thrashing rate for each mutant in the absence (left bar) or presence of doxepinone (right bar) for each mutant. Statistical comparisons were made for each strain in the absence and presence of doxepinone. **(B)** Bar chart showing the reduction in the thrashes/min in each strain due to the presence of doxepinone. ns, non-statistically significant, **p < 0.01 or ***p < 0.001 respect to the change in the wild-type strain.

To determine receptor targets involved in the rapid effects of doxepinone on *C. elegans* swimming rate, we explored the effects on selected mutant strains lacking Cys-loop receptors involved in worm locomotion, which are targets of anthelmintic drugs. The screening is based on the hypothesis that the absence of the target site leads to drug resistance and, therefore, in the mutant worm, the thrashing rate in the presence of doxepinone will not be affected. Since some mutant strains show uncoordinated phenotypes, we determined the number of thrashes/min of wild-type and each mutant strain in liquid medium in the absence and presence of doxepinone (Figure 1A). For each strain, 20 worms in each condition were used, and experiments were repeated with different worm batches and in different days in four independent assays, always in parallel with the control condition.

The thrashing rate of wild-type worms in M9 buffer (plus 1% DMSO) was 204 ± 9.3 min^–1^ (Figure 1A). After 30 min pre-exposure to 0.1 mM doxepinone, this rate decreased about 50% (103 thrashes/min, *p* < 0.001, Student *t*-Test) (Figure 1A).

Mutant worms lacking the UNC-38 subunit (CB904 strain), which is an essential L-AChR subunit, or lacking UNC-49 (GABA) receptors (CB382 strain) showed lower thrashing rates than wild-type worms as well as uncoordinated phenotypes (Brenner, 1974, Figure 1A). Nevertheless, doxepinone reduced the trashing rate in both mutants (Figure 1A, *p* < 0.001), with the magnitude of the reduction being similar to that observed in wild-type worms (Figure 1B). The MT9668 strain lacks the serotonin-gated chloride channel, MOD-1, that is involved in locomotion and behavior (Ranganathan et al., 2000; Komuniecki et al., 2012). This mutant strain was sensitive to the drug (Figure 1A, *p* < 0.001), which produced a decrease of the trashing rate similar to that observed in wild-type worms (Figure 1B). Thus, L-AChR, UNC-49 and MOD-1 receptors are not the main receptors involved in the rapid effects of doxepinone in the swimming rate. The RB918 strain, which lacks the nicotine-sensitive nAChR (N-AChR) present in muscle (Touroutine et al., 2005), was also sensitive to doxepinone (Figure 1A, *n* = 20 worms for each condition). However, the decrease of the trashing rate was slightly, but statistically significantly, lower than that observed for wild-type worms, indicating some type of contribution of this receptor to doxepinone action (Figure 1B, *p* < 0.01).

The thrashing rate of the triple mutant worms lacking three GluCl subunit genes (DA1316) was similar to that of wild-type animals in the absence of the drug (Figure 1A). Interestingly, exposure to 0.1 mM doxepinone did not affect this rate, in contrast to the effects observed in wild-type worms (Figure 1A, *n* = 20, *p* > 0.05). The percentage of the reduction of the thrashing rate due to the presence of doxepinone was statistically significantly different between wild-type and DA1316 worms (Figure 1B, *p* < 0.001). Altogether, our results indicate that GluCls are involved in the rapid effect of doxepinone on the swimming rate.

### 2-Doxepinone-Induced Paralysis Assays on Agar Plates

To determine the type of paralysis and the contribution of the different Cys-loop receptors to doxepinone effects, we performed paralysis assays on agar plates containing the drug.

We first characterized the type of paralysis exerted by doxepinone by exposing wild-type worms to 2.5 mM doxepinone for 30–120 min in agar plates. After 60 min exposure, the worms in the presence of doxepinone were immobile but respond to prodding by contracting body muscle (Supplementary Video S1). After 2 h exposure, worms did not show any response and were completely paralyzed. The length of worms in the absence of the drug was 1.16 ± 0.01 mm whereas it was 1.10 ± 0.04 mm after 1 h exposure (*n* = 10, *p* > 0.05), indicating neither spastic nor flaccid paralysis.

**FIGURE 2:**
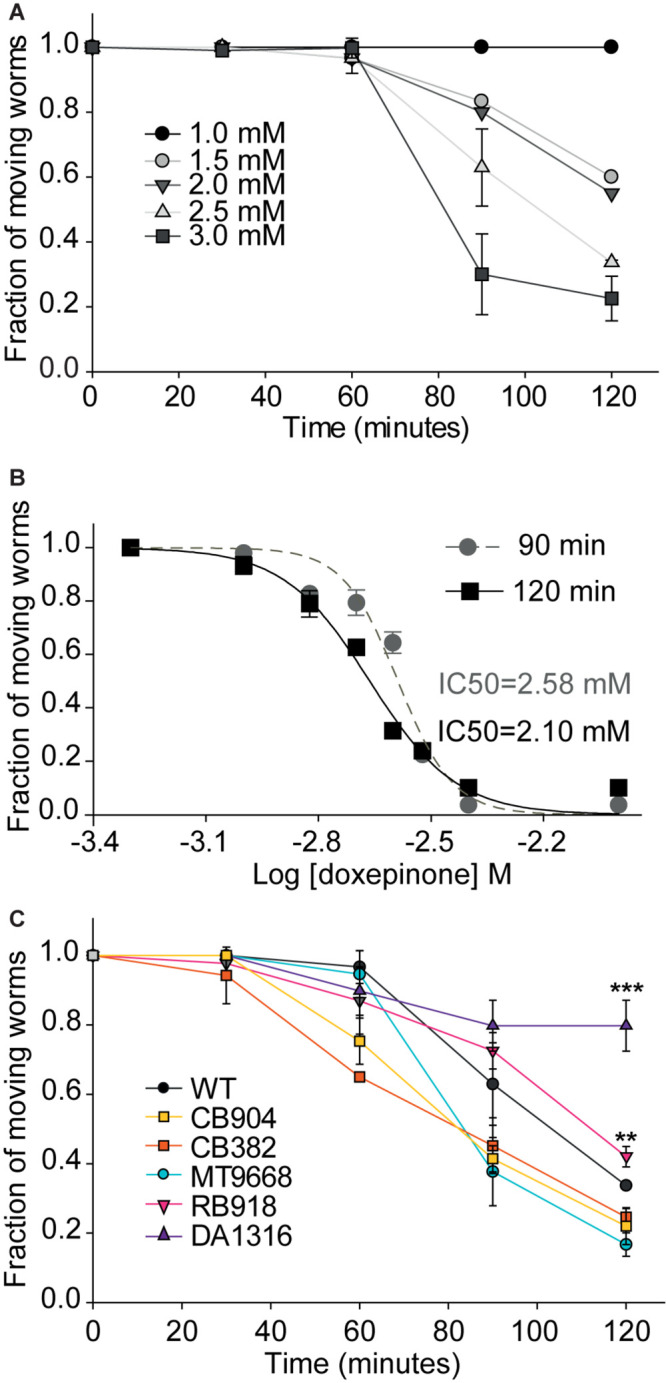
Fraction of moving animals after exposure to doxepinone as a function of time and concentration. Synchronized young adult wild-type worms were placed on agar plates containing DMSO or doxepinone and observed at the indicated time to determine the fraction of worms that respond to prodding, which were considered as “moving worms.” The results correspond to 4 independent assays for all conditions in the figure with 30 worms each time per condition. **(A)** Wild-type worms were exposed in agar plates containing doxepinone (1–3 mM range). The fraction of moving animals was determined at 30 min intervals. **(B)** Dose-response curves determined on agar plates for wild-type worms exposed 90 min (gray) or 120 min (black) to 2.5 mM doxepinone. **(C)** Paralysis as a function of time of exposure of worms to 2.5 mM doxepinone. Statistical comparisons are made respect to wild-type strain. **p < 0.01, ***p < 0.001 respect to the change in the wild-type strain.

For wild-type worms, a clear concentration- and time-dependent paralysis was detected during the 2 h assay at a 1–3 mM doxepinone concentration range (Figure 2A). At the maximum exposure time (2 h), ∼85% adult worms were paralyzed by 3 mM doxepinone (Figure 2A, *n* = 30). The IC_50_ values determined for the inhibition of moving worms in agar plates were 2.58 ± 0.01 mM and 2.10 ± 0.01 mM for 90 and 120 min exposure, respectively (Figure 2B). Paralysis assays on agar plates measured at short times usually require higher drug concentrations than those used in liquid medium probably since the drug must be absorbed from the solid phase.

To confirm the contribution of the different Cys-loop receptors to doxepinone effects, we next measured paralysis as a function of time of different mutant worms exposed to 2.5 mM doxepinone. We found that the reduction of moving worms as a function of time of exposure for MT9668, CB904, and CB382 strains did not differ from that of wild-type worms (Figure 2C). For these mutants, the fraction of moving worms was reduced to ∼0.40 after 2 h exposure on agar plates containing 2.5 mM doxepinone (Figure 2C). Although slight, there was a statistically significant difference in the effects of the drug on RB918 worms (lacking N-AChR) respect to wild-type worms after 2 h exposure (*p* < 0.01, *n* = 30). For worms lacking GluCl subunits (DA1316), only a slight reduction of the fraction of moving animals was detected after 2 h exposure to doxepinone (18%). This reduction was markedly different to that of wild-type worms and other mutants (∼60%), again indicating that GluCls are involved in doxepinone paralysis (Figure 2C).

**FIGURE 3:**
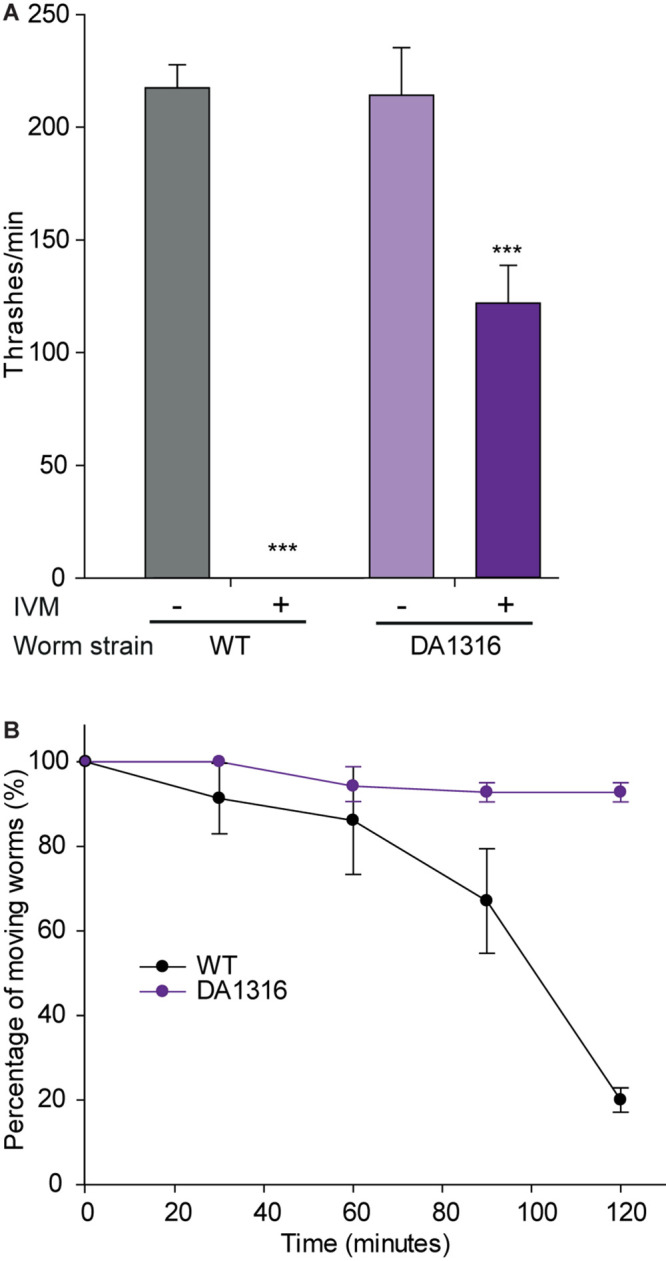
Effects of ivermectin on wild-type and mutant worms lacking GluCl genes. Synchronized young adult worms from wild-type worms or DA1316 strain that lacks GluCl subunit genes. **(A)** Measurements were performed in liquid medium after 30 min incubation in M9 buffer plus M9 buffer containing 1% DMSO in the absence (left bar for each strain) or presence of 10 μM IVM (right bar for each strain). *n* = 20 worms per condition. The results correspond to 4 independent assays of the whole experiment shown in the figure. **(B)** Percentage of moving worms measured on agar plates as a function of time of exposure to 300 μM IVM. Statistical comparisons are made respect to the fraction of wild-type worms. *n* = 20 worms per condition. The results correspond to 4 independent assays of the whole experiment shown in the figure. ***p < 0.001 respect to the change in the wild-type strain.

Because GluCls are targets of IVM we compared the effects of doxepinone with those of IVM on DA1316 (lacking three GluCl genes) and wild-type strains. After 30 min exposure to 10 μM IVM in liquid medium, wild-type worms were fully paralyzed whereas mutant worms (DA1316) showed only 50% reduction of the thrashing rate (Figure 3A). In agar plates containing 300 μM IVM, wild-type animals showed a time-dependent paralysis that yielded 80% paralyzed worms after 2 h. On the contrary, the percentage of paralyzed mutant worms was smaller than 10% after 2 h (Figure 3B). After 2 h exposure, the difference in the fraction of moving worms between wild-type and mutant animals was statistically significative (*p* < 0.001, *n* = 30). We also determined that the type of paralysis of wild-type worms exposed to 300 μM IVM in agar plates during 60 min was similar to that observed for doxepinone: worms appeared immobile but respond to prodding by contracting body muscle (Hernando and Bouzat, 2014; Supplementary Video S2).

### 3-Doxepinone Reduces Pharyngeal Pumping Rate

**FIGURE 4:**
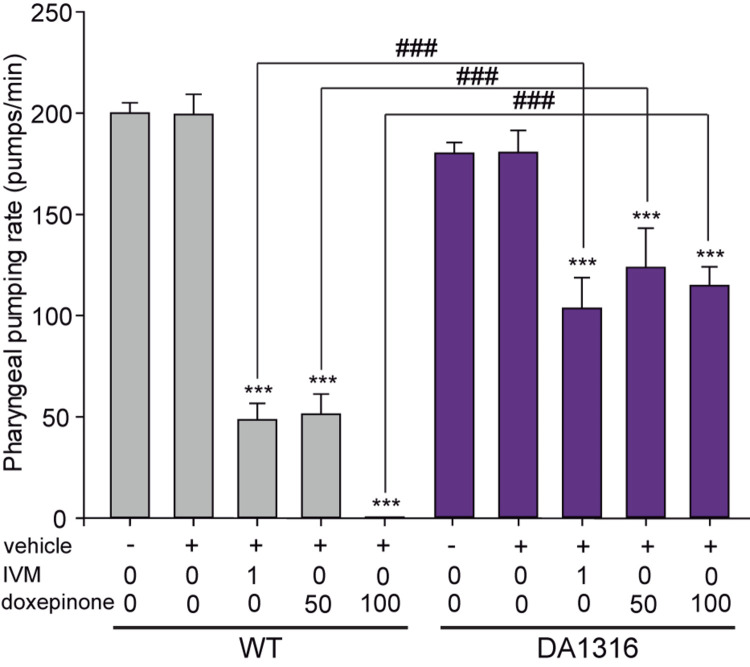
Ivermectin and doxepinone effects on pharyngeal pumping. Wild-type and DA1316 young adult worms were transferred to bacteria-seeded NGM plates containing different concentration of IVM and doxepinone (IVM = 1.0 μM, doxepinone = 50 and 100 μM). After 30 min of drug exposure, the number of contractions in the terminal bulb of the pharynx (pumps) was counted using a stereomicroscope at 50x magnification. Bars represent the mean ± SD from *n* = 14 animals per condition. Statistical differences compared to the non-treated condition of the same strain (***p < 0.001). The symbol # indicates statistically significant differences between the two strains at the same condition (^###^*p* < 0.001).

One of the hallmark effects of IVM involving GluCls is pharyngeal pumping inhibition (Dent et al., 1997, 2000). We therefore evaluated the effects of doxepinone on the pharyngeal pumping rate of wild-type and mutant worms lacking GluCl subunit genes (DA1316). In the absence of drugs, wild-type and DA1316 worms showed similar pumping rates of ∼180–200 min^–1^ (Figure 4). It is important to note that despite lacking GluCls the mutant worms show normal pumping due to compensatory effects and that IVM also affects other Cys-loop receptors (Pemberton et al., 2001; Lynagh and Lynch, 2012; Trojanowski et al., 2016).

The exposure of worms to 1 μM IVM decreased 4-fold the pumping rate in wild-type worms and about 1.7-fold in DA1316 worms (*n* = 14, *p* < 0.001) (Figure 4). These results confirmed that worms lacking GluCl subunits are more resistant to the pumping rate inhibition by IVM than wild-type animals (Figure 4). In wild-type animals, increasing concentrations of doxepinone produced a monotonically decrease of the pharyngeal pumping rate, which was reduced 4-fold at 50 μM and 100% at 100 μM (Figure 4). On the contrary, only ∼1.5-fold reduction was observed in the DA1316 worms in the presence of 100 μM doxepinone with respect to the control, thus indicating that the mutants are resistant to the pharyngeal pumping effect of doxepinone (Figure 4). Thus, the actions of doxepinone correlate with those of IVM and depend on GluCls.

### 4-Molecular Actions of Doxepinone on GluCls

To confirm that doxepinone acts at GluCls and to unravel the mechanism by which it may affect these receptors, we expressed GluClα1/β in BOSC 23 cells and measured whole-cell currents at −60 mV holding potential. Currents were elicited by rapid application of 3 mM glutamate, which is a concentration higher than its EC_50_ for these receptors (∼1.5 mM) but lower than that required for saturation (Cully et al., 1994; Degani-Katzav et al., 2016).

**FIGURE 5:**
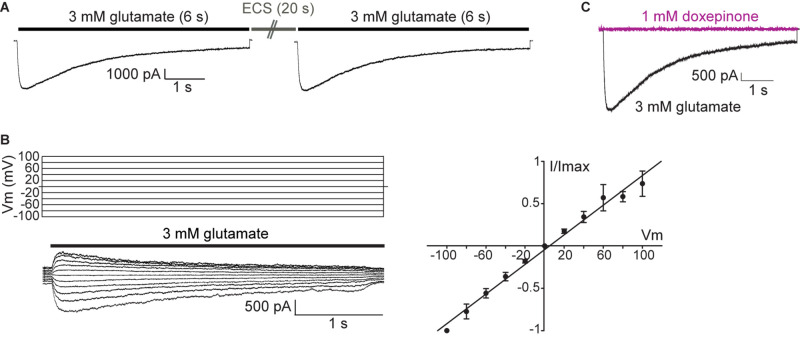
Macroscopic responses of GluClα1/β receptors heterologously expressed in mammalian cells. **(A)** Macroscopic currents elicited by 3 mM glutamate in the whole-cell configuration. Representative responses to glutamate in a single cell. Cells were first exposed to a 6-s pulse of 3 mM glutamate, then to a 20-s pulse of ECS alone, and finally again to the 3 mM glutamate-containing ECS. Pipette potential: -60 mV. **(B)** Left: currents elicited by 6 s -pulse of 3 mM glutamate at different pipette potentials (from 100 to -100 mV, Vm). Right: Current (I)-Voltage (Vm) relationship for GluCl channels under symmetrical chloride solutions (*n* = 4). **(C)** Cells were first exposed to a 6-s pulse of 3 mM glutamate (black line current), then to a 6-s pulse of 1 mM doxepinone (violet line current), and finally again to the 3 mM glutamate-containing ECS to verify current recovery. Pipette potential: −60 mV.

A 6-s pulse of 3 mM glutamate in ECS elicited macroscopic currents that reached the peak with a rise time of 49.30 ± 16.20 ms (*n* = 9) and decayed in the presence of the agonist due to desensitization. Typically, peak currents varied between 2000 and 7000 pA. The decay was fitted by a single component with a time constant of 2100 ± 1040 ms (*n* = 9). The application of another pulse of glutamate after a 20-s wash with ECS allowed full recovery of the peak current, indicating that most receptors recovered from desensitization in the absence of the agonist after this period (Figure 5A).

To further characterize GluCl-mediated currents, we constructed current-voltage relationships by measuring the peak current elicited by 3 mM glutamate as a function of the holding potential (Figure 5B). As shown in the figure, the magnitude of GluCl-elicited currents increased linearly with the voltage, indicating an ohmic behavior, and currents did not show important rectification (*n* = 4).

We next proceeded to decipher the molecular actions of doxepinone at GluCls. To first determine if doxepinone can activate GluCls, a 6-s pulse of 0.5 mM doxepinone (*n* = 7) or 1 mM doxepinone (*n* = 3) was applied to cells in the whole-cell configuration at -60 mV. No currents were elicited by doxepinone and sequential application of 3 mM glutamate to the same cell elicited macroscopic responses, indicating the presence of functional GluCls (Figure 5C). Thus, doxepinone does not act as an agonist of GluCls.

We next evaluated the action of doxepinone as a modulator of glutamate-activated currents. To this end, we used two different drug application protocols, one including preincubation of the drug before glutamate application (Preincubation protocol) and the other, application of doxepinone together with glutamate (Co-application protocol).

**FIGURE 6:**
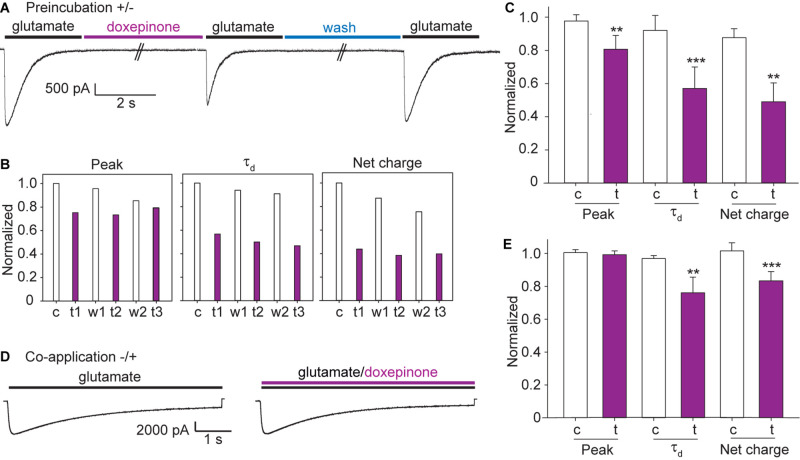
Effects of doxepinone on glutamate-elicited responses using different application protocols. Doxepinone (1 mM) was applied before the 3 mM glutamate pulse (preincubation, ± protocol) or together with glutamate (co-application, −/ + protocol). Pipette potential: −60 mV. **(A)** ± protocol: 6 s-pulse of 3 mM glutamate was applied before (control current, c) and after (treated current, t) preincubation during 60 s with 1 mM doxepinone. Currents were recorded after 1 min wash with ECS to confirm recovery (recovered, w). This protocol was repeated 3 times in each cell. **(B)** Illustrative example of the changes in the macroscopic current parameters obtained after each application of 3 mM glutamate in a single cell with the protocol shown in **(A)**. c, control current; t and w, correspond to the glutamate-activated current obtained after 60-s incubation with 1 mM doxepinone (treated, t) or buffer alone (wash, w). The sub index corresponds to the agonist-application order in the series. **(C)** Bar chart showing the averaged changes in peak current, decay time constant and net charge due to the preincubation with 1 mM doxepinone (t). For each experiment, the peak current, the decay time constant and total area were related to those of the control current in each cell (c). The values correspond to the mean of 6 different cells and 4 different days of transfection (***p < 0.001; **p < 0.01). **(D)** −/ + protocol: 6 s-pulse of 3 mM glutamate was first applied alone (control current, black line) and then together with 1 mM doxepinone (treated current, violet line). **(E)** Bar chart showing the effects of 3 mM glutamate/1 mM doxepinone co-application on peak current, decay time constant and net charge. The values correspond to the mean ± SD of 5 different cells (***p < 0.001; **p < 0.01; *p < 0.05).

For the preincubation protocol, a pulse of ECS containing 3 mM glutamate (6 s-pulse, control current) was first applied to the cell held at -60 mV, and the cell was incubated during 1 min with ECS containing doxepinone (1 mM) before a second pulse of ECS-glutamate was applied (treated) (Figure 6A). The same protocol was repeated three times in the same cell, each time separated by a 60-s period. An illustrative example of an experiment from a single cell is shown in Figures 6A,B. The results from different cells were averaged and shown in Figure 6C.

Preincubation of the cell with 1 mM doxepinone produced a slight decrease of the peak current and a profound decrease of the current decay time constant and net charge (Figure 6C). Compared to the corresponding control current in each cell, the peak current was reduced to 0.81 ± 0.08 (*p* = 0.001), the decay rate to 0.57 ± 0.13 (*p* = 0.001), and the net charge to 0.49 ± 0.11 (*p* = 0.002, *n* = 6). Thus, the main effect of the drug on glutamate-elicited currents is the increase in the decay rate (or decrease of the decay time constant), which leads to a concomitant decrease in the net charge. We also determined that the percentage of changes after drug application were similar among the three applications on each cell. Also, no significant differences were found in the relative peak current (1.01 ± 0.10), net charge (0.99 ± 0.17), and decay time constant (0.95 ± 0.06) with respect to the control when preincubation was performed with 0.2% DMSO in ECS in the absence of doxepinone (*n* = 4).

We also explored if doxepinone applied together with glutamate (co-application protocol) affected GluCl currents (Figures 6E,D). In each cell, we applied three pulses of glutamate containing ECS, each one separated by 20 s, and then three pulses of ECS containing 3 mM glutamate and 1 mM doxepinone (Figure 6D). Doxepinone did not produce statistically significant changes in the peak currents (0.99 ± 0.02, *p* = 0.319) but produced a slight and statistically significantly decrease of the net charge (0.83 ± 0.06, *p* = 0.001) and decay time constant (0.76 ± 0.10, *p* = 0.008) (*n* = 5 cells) (Figure 6E). Thus, co-application of doxepinone inhibited glutamate-activated currents but the changes were smaller than those determined under the preincubation protocol.

## Discussion

We here identified dibenzo[*b,e*]oxepin-11(6H)-one (doxepinone) as a novel anthelmintic compound acting through GluCls by a different mechanism to that of the widely used anthelmintic drug, IVM, and proposed that GluCl inhibition may be further explored as a mechanism of action of anthelmintic drugs.

The anthelmintic action of doxepinone is revealed by the induction of worm paralysis measured in agar plates, the inhibition of worm mobility in liquid medium, and an important decrease of the pumping rate. Because rapid effects as the ones observed for doxepinone may be mediated by ion channels, we performed the first screening for resistance to doxepinone on selected null mutant strains lacking Cys-loop receptors involved in worm locomotion. The screening identified GluCls, which are main receptor targets of IVM, as targets of doxepinone. In close agreement with this finding, we demonstrated that the effects of doxepinone recapitulate those of IVM. Particularly, the inhibition of the pumping rate is a hallmark of IVM action. Also, the paralysis induced by doxepinone is neither spastic nor flaccid as that observed in the presence of IVM.

Our screening showed that N-AChRs may play a role in the paralysis caused by doxepinone since the worms lacking the ACR-16 subunit are less sensitive to the drug than wild-type worms. However, the effect is not as relevant as that mediated by GluCls. Also, we cannot discard that other receptors, not explored here, may be involved in doxepinone effects on *C. elegans*. Finally, it would be also interesting to perform studies on parasitic nematodes to confirm that they respond to doxepinone similarly to the free-living nematode.

Inhibitory GluCls are expressed on neurons and muscle across protostome phyla, including mollusks, flatworms, nematodes, ticks, and mites, as well as insects and crustaceans (Wolstenholme, 2012). They are of great importance since they are one of the main target sites for parasitic control. IVM and macrocyclic lactones are used to eliminate nematode infections from millions of humans suffering from diseases, such as river blindness and lymphatic filariasis, and are also used in domestic pets and cattle for parasitic nematodes and ectoparasites (Omura, 2008). GluCls in *Anopheles gambiae sensu stricto* were also proposed as targets of IVM to control malaria (Meyers et al., 2015; Atif et al., 2019).

In *C. elegans*, the expression pattern of the different GluCl genes has been explored (Holden-Dye and Walker, 2014). In particular, GluClα1 subunit expresses in body wall muscle, head neurons, and pharyngeal muscle cells and GluClβ subunit in pharyngeal muscle pm4 cells. Although GluCl subunits can form homomeric or heteromeric receptors in heterologous expression systems, the composition of the native receptors remains mostly unknown. To elucidate the molecular functional consequences of doxepinone acting at GluCls, we used as a model the GluClα1/β receptor, which has been previously characterized in detail in mammalian cells (Degani-Katzav et al., 2016). Since the DA1316 strain lacks three GluCl genes, *avr-14, avr-15*, and *glc-1*, it would be interesting to explore in future work the molecular effects of doxepinone on other GluCl subtypes (Brockie and Maricq, 2006).

Cells expressing GluClα1/β receptors displayed robust responses to 3 mM glutamate, in line with previous findings (Degani-Katzav et al., 2016). By analyzing the peak current as a function of voltage we determined that the ion channel has an ohmic behavior with no significant rectification.

Currents could not be elicited by the solely application of doxepinone, indicating that it is not an agonist of this type of GluCl, an action distinct from that exerted by IVM. Pre-exposure of GluCls to doxepinone before activation with the agonist significantly decreased the net charge, thus revealing that doxepinone acts as an inhibitor. The analysis of current parameters showed a slight decrease of the peak current and a significant increase of the current decay rate, indicating that this latter change governs the negative modulation. The changes are qualitatively similar but quantitatively smaller when doxepinone is applied together with glutamate with no preincubation. Receptor inhibition may be caused by competitive or non-competitive antagonism. If inhibition were due to competitive antagonism, a reduction of the peak current instead of an increase in the decay rate would be observed. Also, the effects produced by co-application of doxepinone with glutamate would be greater than those observed with preincubation, which is opposite to our experimental results. Thus, our first molecular characterization suggests that doxepinone acts as a negative allosteric modulator (non-competitive antagonist) of GluCl. The enhancement of the current decay rate due to the presence of allosteric inhibitors may arise from enhanced desensitization or channel block (Gumilar et al., 2003). However, further electrophysiological characterization, including competition studies, is required to unequivocally define its mechanism of action as well as its potential binding sites at GluCl.

The three-dimensional atomic structure of the homomeric *C. elegans* GluClα shows that IVM occupies a cavity between adjacent subunits in the transmembrane domain (Hibbs and Gouaux, 2011). Further studies combining mutant receptors may help to define if doxepinone binds to the IVM site.

Although sharing the target receptor, IVM and doxepinone are non-related, structurally different compounds. Whereas IVM is a high molecular weight macrocyclic lactone derived from avermectins, doxepinone constitutes a class of fused tricyclic heterocycles present in a variety of bioactive compounds. Structurally, it is typified by the presence of a dibenzo-4-oxepanone ring system. Each ring is connected in a fused formation that does not allow rotation around the carbon-carbon bonds. This unique structure together with the type and position of the linked chemical groups define the specific functionalities of doxepinone. The hydrophobic planar architecture of this oxygenated heterocyclic gives it a remarkably different physicochemical behavior with respect to IVM.

The structure of doxepinone is related to that of tricyclic antidepressants, in particular to doxepin. Tricyclic antidepressants have been shown to inhibit other Cys-loop receptors, including vertebrate nAChR and 5-HT_3_A receptors (Gumilar et al., 2003; Gumilar and Bouzat, 2008). Doxepin reduced the peak current and increased the decay rate of mouse muscle nAChRs; these effects were greater when applied during preincubation than co-applied with the agonist (Gumilar et al., 2003). Enhancement of desensitization and/or slow channel blockade was proposed as the mechanism underlying the macroscopic effects (Gumilar et al., 2003). Although we have shown previously that doxepin slightly decreased the thrashing rate in *C. elegans*, the effect was significantly lower compared to that of doxepinone (Scoccia et al., 2017). Interesting, it has been reported that doxepin shows anthelmintic activity against the intestinal helminth *Ancylostoma ceylanicum*, revealing an antiparasitic action (Keiser et al., 2016).

The widely use of IVM has resulted in selection of resistant parasitic nematodes, which has turned into a major global problem (Laing et al., 2017). It has also raised concerns that IVM resistance may evolve in human parasites as well (Laing et al., 2017). Mechanisms underlying IVM resistance include changes in sequence and composition of GluCls and in proteins regulating membrane permeability and gap junctions. Importantly associated to IVM resistance is the enhanced expression of the multidrug transport protein, P-glycoprotein, involved in drug exclusion (Blackhall et al., 1998; Xu et al., 1998; Le Jambre et al., 1999; Sangster et al., 1999; Ardelli and Prichard, 2013; Ménez et al., 2016). Since IVM and doxepinone are structurally different compounds, they will probably show different activities at P-glycoprotein as well as different sensitivities among GluCl subtypes. Thus, our finding offers a new scaffold for developing new GluCl-active compounds directed to overcome IVM resistance as well as to cover different helminth species.

The effect of IVM and macrocyclic lactones has been proposed to be mediated by increased hyperpolarization due to its agonistic activity at chloride permeable GluCls. We here found that doxepinone has the opposite effect. Therefore, the inhibition of GluCls emerges as an anthelmintic mechanism of action. In line with this, for several insecticides, such as picrotoxin, lindane, and fipronil, the inhibition through GluCl has been proposed as a mechanism involved in their insecticide actions (Narahashi et al., 2010; Atif et al., 2019). Fipronil has been shown to reversibly inhibit GluCls from *C. elegans* (Horoszok et al., 2001) and from *Haemonchus contortus* (McCavera et al., 2009) and it has been shown to be effective for controlling nematodes on wheat (Cui et al., 2017). Thus, it appears that both enhanced and reduced hyperpolarization can affect worm locomotion and pharyngeal pumping. How neuron wiring underlying the behavioral effects is affected as a result of reduced hyperpolarization due to GluCl inhibition is therefore an essential question for future studies. Overall, we propose doxepinone as a new scaffold with potential antiparasitic activity and the inhibition of GluCls as a mechanism of anthelmintic drug action valuable to be further explored.

## Data Availability Statement

All datasets generated for this study are included in the article/Supplementary Material.

## Author Contributions

MC, OT, MF, DG, and CB contributed to study design. MC and OT acquisition of data. MC, OT, DG, and CB analysis and interpretation of data and contributed to writing. All authors contributed to the article and approved the submitted version.

## Conflict of Interest

The authors declare that the research was conducted in the absence of any commercial or financial relationships that could be construed as a potential conflict of interest.

## Footnotes

1 www.wormbook.org

## Supplementary Material

FIGURE S1 Total synthesis of doxepinone. Conditions: **(A)** NaH (1.5 equiv), DMF (dimethylformamide), reflux, 24 h; then conc. HCl; **(B)** FeCl_2_ (0.6 equiv), DCME (dichloromethyl methyl ether) (1 equiv), DCM (dichloromethane) (0.1 M), rt. Isolated yield (%) after purification. The details of the procedure have been described in Scoccia et al. (2017).

VIDEO S1 Wild-type worms exposed to 2.5 mM doxepinone in agar plates. The worms are immobile but respond to prodding by contracting body muscles.

VIDEO S2 Wild-type worms exposed to 300 μM IVM in agar plates. The worms are immobile but respond to prodding by contracting body muscles.

## Abbreviations

ECS: extracellular solution
GluCl: glutamate-gated chloride channel
ICS: intracellular solution
IVM: ivermectin
L-AChR: levamisole-sensitive acetylcholine receptor
N-AChR: nicotine-sensitive acetylcholine receptor
nAChR: nicotinic acetylcholine receptor
NGM: nematode growth medium
τ_*d*_: current decay time constant.
