# Supplementary material for: A New Antagonist of Caenorhabditis elegans Glutamate-Activated Chloride Channels With Anthelmintic Activity
Source: Front Neurosci. 2020 Aug 19;14:879. doi: 10.3389/fnins.2020.00879 (PMC7466757; doi:10.3389/fnins.2020.00879)
Supplement: FIGURE S1 — Total synthesis of doxepinone. Conditions: (A) NaH (1.5 equiv), DMF (dimethylformamide), reflux, 24 h; then conc. HCl; (B) FeCl2 (0.6 equiv), DCME (dichloromethyl methyl ether) (1 equiv), DCM (dichloromethane) (0.1 M), rt. Isolated yield (%) after purification. The details of the procedure have been described in Scoccia et al. (2017). [file Presentation_1.pdf]

**SUPPLEMENTARY MATERIAL**

# A new antagonist of *Caenorhabditis elegans* glutamate-activated chloride channels with anthelmintic activity

**María Julia Castro[1,2&], Ornella Turani[1&], María Belén Faraoni[2], Darío Gerbino[2] and Cecilia Bouzat[1*]**

[1]Instituto de Investigaciones Bioquímicas de Bahía Blanca (INIBIBB), Departamento de Biología, Bioquímica y Farmacia, Universidad Nacional del Sur (UNS)-Consejo Nacional de Investigaciones Científicas y Técnicas (CONICET), 8000 Bahía Blanca, Argentina.

[2]Instituto de Química del Sur (INQUISUR), Universidad Nacional del Sur (UNS)-Consejo Nacional de Investigaciones Científicas y Técnicas (CONICET), 8000 Bahía Blanca, Argentina.

**Supplementary Figure 1**.

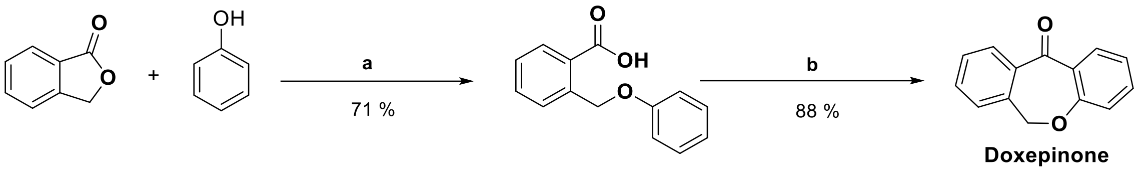


Total synthesis of doxepinone. Conditions: a) NaH (1.5 equiv), DMF (dimethylformamide), reflux, 24 h; then conc. HCl; b) $FeCl_2$ (0.6 equiv), DCME (dichloromethyl methyl ether) (1 equiv), DCM (dichloromethane) (0.1 M), rt. Isolated yield (%) after purification.
The details of the procedure have been described in Scoccia et al. (2017).
